# Immediate implant placement in molar extraction sockets: a systematic review and meta-analysis

**DOI:** 10.1186/s40729-020-00235-5

**Published:** 2020-08-08

**Authors:** Gian Maria Ragucci, Basel Elnayef, Elena Criado-Cámara, Fernando Suárez-López Del Amo, Federico Hernández-Alfaro

**Affiliations:** 1 Department of Oral and Maxillofacial Surgery, International University of Catalonia, Josep Trueta, s/n, 08195 Sant Cugat del Vallès, Barcelona, Spain; 2 Tacoma, WA USA

## Abstract

**Background:**

Immediate implants are frequently employed in the anterior maxillary area. However, the installation of dental implants simultaneously with tooth extraction can also provide with benefits in the posterior areas with a reduction in time prior the recovery of the masticatory function. Results previously reported in the literature show high-survival and success rates for implants placed in extraction sockets in molar areas; however, this topic has received limited systematic analysis.

**Material and methods:**

Electronic and manual literature searches were performed by two independent reviewers in several data-bases, including MEDLINE, EMBASE, and Cochrane Oral Health Group Trials Register, for articles up to January 2019 reporting outcomes of immediate implants placed in molar areas. Primary outcomes included survival and success rates, as well as marginal bone loss. Secondary outcomes included the influence of implant position, type of implant connection, grafting protocol, flap or flapless approach, implant diameter, surgical phase, presence of buccal plate, and loading protocol.

**Results:**

Twenty studies provided information on the survival rate, with a total sample of 1.106 implants. The weighted mean survival rate of immediate implants after 1 year of follow-up was 96.6%, and the success rate was 93.3%. On the other hand, marginal bone loss was 1.29 ± 0.24 mm. Secondary outcomes demonstrated that grafting the gap and the loading protocol have an effect on survival and success rates. Similarly, the presence or absence of the buccal bone affect crestal bone levels. Meta-analysis of 4 investigations showed a weighted mean difference of 0.31 mm ± 0.8 IC 95% (0.15–0.46) more marginal bone loss at immediate implant placement versus implants in healed sites (*p* < 0.001) *I*^2^ = 15.2%.

**Conclusion:**

In selected scenarios, immediate implant placement in molar extraction socket might be considered a predictable technique as demonstrated by a high survival and success rates, with minimal marginal bone loss.

## Introduction

As implant therapy evolves, new challenges are faced as result of higher functional and esthetics demands. Original protocols during the late 1970s and 1980s advocated the placement of dental implants exclusively in completely healed edentulous ridges. Additionally, a healing period of 6–12 months before loading was recommended [1].

Fortunately, as a consequence of continuous research, new surface technologies, and new implant designs, more recent protocols have arisen shortening the time intervals by means of immediate implant placement (IIP) and/or immediate restorations (IR). Although different definitions have been proposed for the terms immediate, early and conventional implant placement, in 2004, a consensus statement by Hämmerle and coworkers described 4 different protocols for implant placement in the extraction socket. Type 1 refers to IIP after tooth extraction, type 2 is considered when implants are placed after 4 to 8 weeks, achieving complete soft tissue coverage, type 3 consists in implants placed after 12 to 16 weeks when substantial clinical and/or radiographic bone fill has occurred, and type 4 refers to implants placed in healed sites [2].

IIP is a therapeutic approach introduced in 1976 as an alternative to the classic delayed implant placement described by Branemark [3]. This treatment alternative offers several advantages; including a reduction in both treatment time and the number of surgical interventions, therefore increasing patient satisfaction. In addition to these advantages, survival and success rates of immediate implants have shown favorable results, proving similar outcomes to implants installed in healed edentulous ridges. A systematic review on implants installed immediately after tooth extraction demonstrated survival rates of more than 98% after a minimum of 1-year follow up. These results are comparable to conventional implant placement in healed sites which showed 5-year survival rates of up to 95% [4]. This is also in agreement with a randomized controlled clinical trial that demonstrated adequate hard and soft tissue healing with stable marginal bone levels after 3 years of follow-up for immediate implants placed in the anterior area [5]. The esthetic advantages of IIP in combination with IR for patients that have lost anterior teeth becomes obvious when treatment time is reduced; decreasing the waiting period prior delivery of a fixed restoration. However, IIP in posterior areas may also represent a beneficial approach in selected scenarios providing with a faster recovery of the masticatory function.

Results reported in the literature have shown high survival (99.1–100) and success rates (93.9–100%) for implants placed in extraction sockets on molar areas. Similarly, a systematic review published in 2010 reported up to 99% survival rate for implants placed in posterior areas. Nevertheless, multitude of new investigations have emerged since this review was conducted. These recent studies could offer further information on the outcomes and considerations for this treatment alternative [6–12].

The aim of this systematic review was to assess the survival and success rates, as well as the marginal bone loss (MBL) of IIP in molars extraction sockets after a minimum follow up of 1 year.

## Material and methods

### Search strategy

Three electronic databases were used including PubMed, Ovid (MEDLINE), and Cochrane Central for relevant studies published in the English language without any time limitation. The search was conducted up to January 2019 by two independent examiners (G.M.R and B.E) aiming at answering the following PICO (Patient, Intervention, Comparison and Outcome) question: In patients over 18 years of age, does the placement of immediate implants in molar areas result in similar implant survival rate, success rate, and marginal bone loss as implants installed in healed sites, after 6 months of healing from tooth extraction? When necessary, disagreements were resolved by discussion with a third examiner (F.H.A). Search terms included “Jaw, edentulous”[mh] OR “Alveolar process”[mh] OR “Alveolar bone loss”[mh] OR “Dental implantation”[mh] OR “Dental implants”[mh] OR “Dental prosthesis design”[mh] OR “Denture”[mh] OR “Dental prosthesis, implant-supported”[mh] OR “molar”[tiab] OR “Fresh socket”[tiab] AND (“Immediate”[tiab] OR “Immediate non-occlusal”[tiab] OR “Functional”[tiab] OR “Non-functional”[tiab]) AND (“Provisionalization”[tiab] OR “Restoration”[tiab] OR “Loading”[tiab])

In addition, a review of the references of the included investigations was performed. Finally, hand search (Jan 2000–Jan 2019) was carried out in dental journals, including *Journal of Oral and Maxillofacial Implants, Clinical Implant Dentistry and Related Research*, *Clinical Oral Implants Research, Implant Dentistry*, *European Journal of Oral Implantology*, *Journal of Oral Implantology*, *International Journal of Oral and Maxillofacial Surgery*, *Journal of Oral and Maxillofacial Surgery*, *Journal of Dental Research*, *International Journal of Prosthodontics*, *Journal of Prosthetic Dentistry*, *Journal of Clinical Periodontology*, *Journal of Periodontology*, and *The International Journal of Periodontics and Restorative Dentistry*.

Articles were included if they met the following inclusion criteria: human randomized controlled trials, prospective cohort studies, retrospective studies, and case series with a minimum of 10 subjects; studies with at least 1 year of follow-up; studies reporting data on marginal bone loss and survival rates of immediate implants placed in molar sites. On the other hand, articles were excluded if they present with any of the following characteristics: implants placed following early protocol; unknown survival rate, success rate or marginal bone loss; less than 1 year of follow-up; less than 10 subjects for the immediate implant group, interventions involving simultaneous lateral or crestal sinus floor elevation, inferior nerve transposition or sandwich osteotomy, animal studies, and implant placement in non-molar areas.

Primary outcomes in this systematic review included (1) *survival rate (*defined as implant present in the oral cavity independent of biological or technical complications), (2) success rate (defined as implants free of all complications); and (3) marginal bone loss. Secondary outcomes included: implant position, type of implant connection, grafting protocol, flap or flapless approach, implant diameter, surgical phase, presence or absence of buccal plate, and loading protocol.

### Selection of studies

Two independent reviewers (G.M.R and B.E) screened all titles and determined the number of abstracts to be evaluated. All selected abstracts were screened for possible inclusion in the systematic review. The full texts of all studies of relevance were then obtained for independent assessment by the reviewers, and any disagreement was resolved by discussion with a third examiner.

### Quality assessment

The criteria used to evaluate the quality of the selected randomized controlled trials (RCTs) were modified from the randomized clinical trial checklist of the Cochrane Center and the CONSORT (Consolidated Standards of Reporting Trials) statement, which provided guidelines for the following parameters: (1) sequence generation; (2) allocation concealment method; (3) masking of the examiner; (4) address of incomplete outcome data; and (5) free of selective outcome reporting. Two independent reviewers (G.M.R and B.E) evaluated all the included articles. On the other hand, for non-randomized clinical trials, the Newcastle-Ottawa Scale (NOS) was used to rank risk of bias of included studies.

### Statistical analysis

The R 3.0.2 software package was used to perform the meta-analysis. The pooled weighted mean (WM) and the 95% confidence interval (IC) of each variable were estimated using a computer program (Comprehensive Meta-analysis version 2, Biostat). Random effects meta-analyses of the selected studies were applied to account for potential bias arising from methodology. The analysis consists in the estimation of the survival and success rates, as well as the weighted average MBL for the whole of the studies, using a random effects model. Estimates of mean proportions and bone loss, both individual, for each investigation and global, are accompanied by the 95% confidence interval and are represented by a Forest graph.

### Meta-analysis

For the study of the survival rate, odds ratios were estimated for each study, as well as the overall effect measure in a meta-analysis of random effects, always with 95% confidence intervals. For the analysis of MBL, the difference between the average value of the test group: immediate implants in molar extraction socket, and the control group: implants in healed sites, after 6 months of healing from tooth extraction were calculated. The weighted mean difference (WMD) was the overall effect measure, estimated by a random effects meta-analysis.

### Study of heterogeneity

Heterogeneity was assessed based on calculation of the *I*^2^ statistic (percentage variability of estimated effect that can be attributed to the heterogeneity of the effects) and the null statistic test. Galbraith graphs displayed the degree of heterogeneity. Funnel plots and the Egger test were used to assess risk of bias of the accepted statistical significance level was 5% (*p* = 0.05).

## Results

**Fig. 1:**
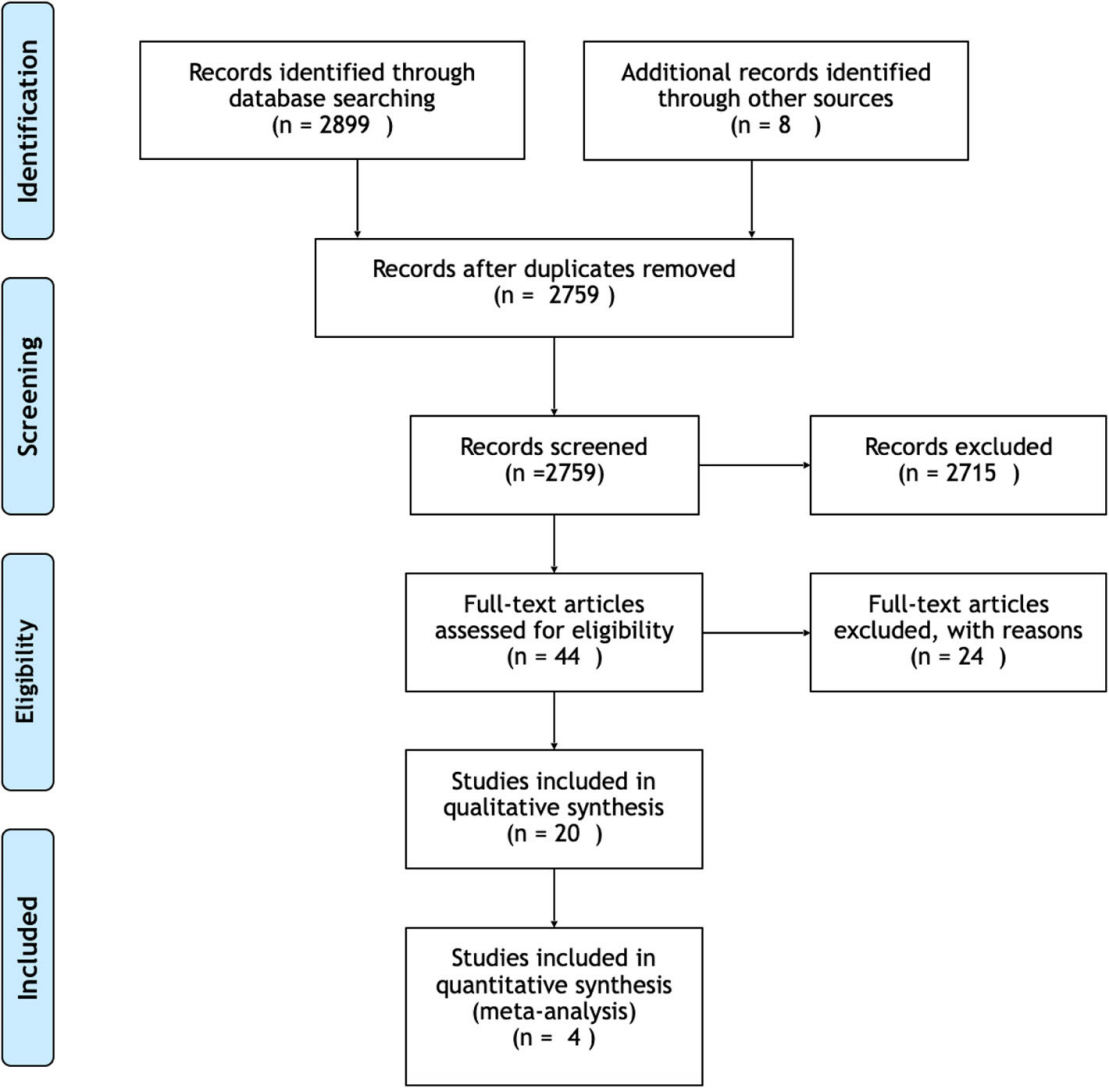
PISMA flowchart of the screening process

The search resulted in 2759 titles. Following the first stage of screening, 44 potentially relevant studies were identified. After the second stage of screening, full text publications were obtained and analyzed, resulting in 20 articles fulfilling the inclusion criteria (Fig. 1). Reasons for exclusion of articles after full text analysis were: absence of report of data on MBL, studies with less than 10 subject, studies on implants placed in the anterior zone, studies that included anterior and posterior implants within the same groups, and studies that failed to specify timing of implant placement (Table 1). All of the included investigations had a follow up of at least 1 year; 7 studies reported a follow-up of more than 18 months and one study showed an observational period up to 5 years. The majority of studies were conducted in an institutional environment. A total of 990 patients were analyzed in this review, including 1.106 implants Table 2.

**Table 1:** Articles excluded and reasons for exclusion

Reason for exclusion	Study
No report on data of MBL	Ormanier et al. 2012, Carlino et al. 2008, Acocella et al. 2010
Less than 10 subjects included	Youself et al. 2012, Block et al. 2011
Implants placed in anterior area	De Angelis et al. 2011, Gómez Roman et al. 2001, Paoloantonio et al. 2001, Harel et al. 2014, McAllister et al. 2012, Malchiodi et al. 2010, Siepnkothen et al. 2007
Implant placement timing not specified.	Cavallaro et al. 2011

**Table 2:**
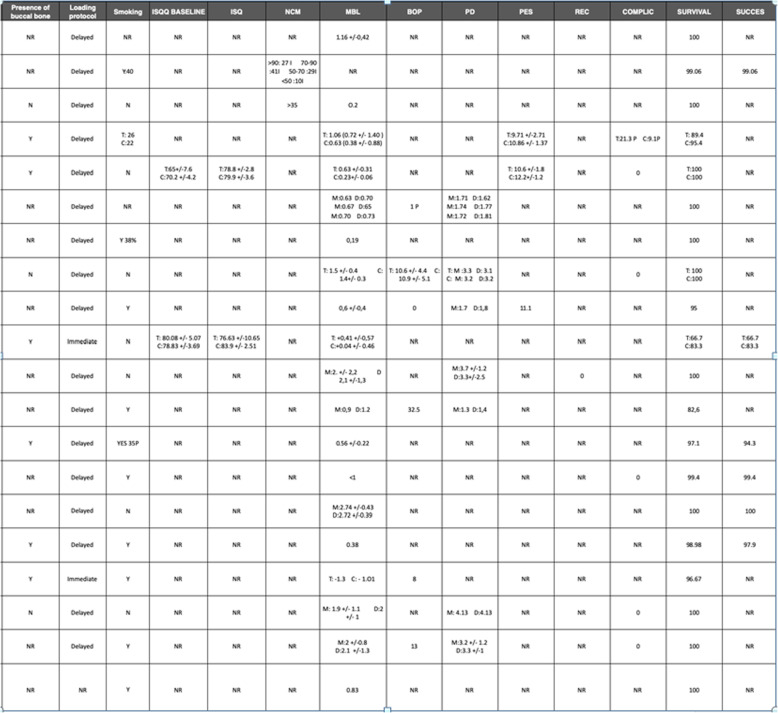
Characteristics of the included articles

Primary outcomes in this systematic review included *survival*, *success rates*, and *MBL*. Secondary outcomes included the analysis of the influence of: implant position, type of connection, grafting protocol, flap or flawless surgery, implant diameter, surgical phase, presence of buccal plate, and loading protocols.

### Implant survival

**Fig. 2:**
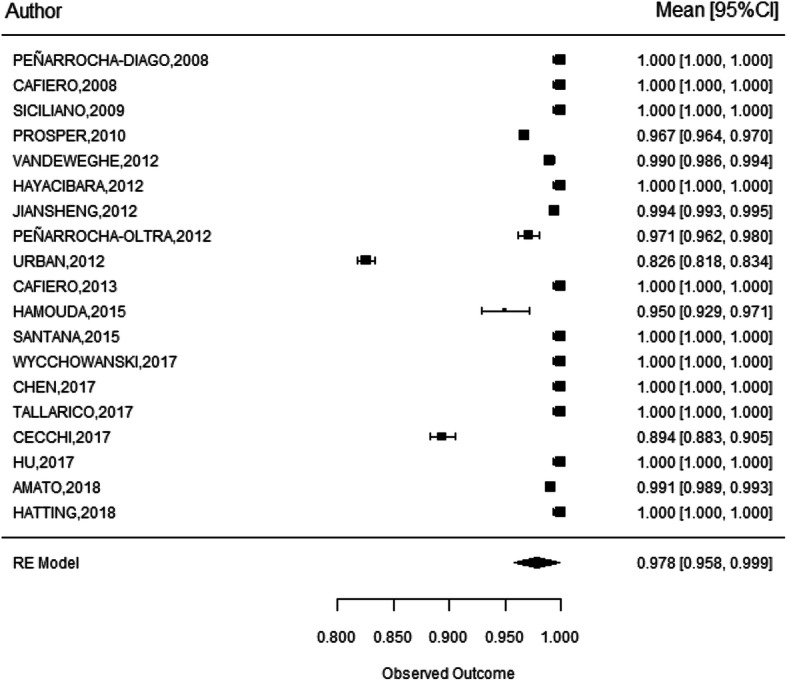
Statistical analysis of implant survival

Survival was defined as implants remaining in situ at the follow-up examinations, irrespective of their conditions. All 20 studies reported survival rates, leading to a weighted mean survival rate of 96.6% with 95% CI (93.5–99.7) [13–32]. Certain considerations must be taken into account when interpreting the estimates of the individual studies: the study by Atieh et al. shows a greater standard deviation, due to the small sample size, only 12 implants [13]. For this reason, it is considered appropriate to exclude this study from the meta-analysis. The model is re-estimated, obtaining: a weighted survival rate of 97.8% with an IC 95% (95.8-99.9) (Fig. 2)

### Implant success

**Fig. 3:**
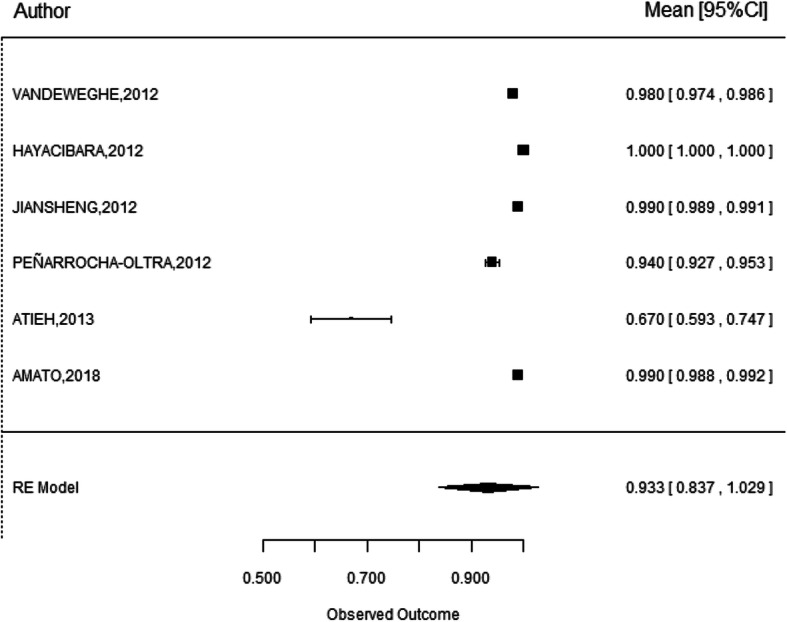
Statistical analysis of implant success

The success rate was only analyzed in 6 studies [13, 16, 20, 22, 28, 30] leading to a weighted mean success rate of 93.3% with 95% CI (83.7–100). Excluding again the study of Atieh, the success rate increases to 98.1% with 95% CI (96.1–100) (Fig. 3) [16, 20, 22, 28, 30].

### Marginal bone loss

**Fig. 4:**
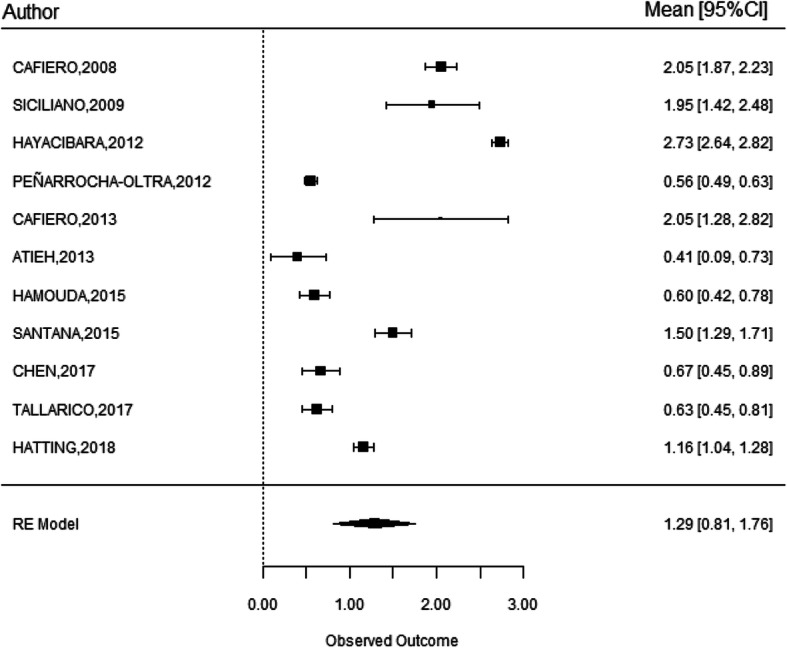
Statistical analysis of marginal bone loss

Eleven studies reported on MBL, analyzed through the use of periapical radiography, including data of 372 implants [13, 16, 22, 18–21, 24, 25, 27, 29, 32]. The estimated global MBL over 1 year of follow-up was 1.29 ± 0.24 mm with 95% CI (0.81–1.76) (Fig. 4).

### Implant position

Six investigations were included for the analysis of implant position, four studies reported implants placed in the mandible [13, 14, 19, 20], two studies reported implants placed in maxilla [25, 26]. Studies assessing implant survival in the posterior maxilla yielded a 100% survival rate. Implant survival in the posterior mandible yielded a 97.4% survival rate and an 97.5% success rate. No statistically significant difference in survival and success rates were detected according to the implant position (*p* = 0.233). MBL assessed according to implant location, also revealed no significant differences when comparing maxilla and mandible (*p* = 0.991).

### Type of implant-abutment connection

The studies included in this systematic review that analyzed survival and success rates as well as MBL depending on the type of implant-abutment connection [13, 15–32] reported no statistically significant for implant survival rate (*p* = 0.511) success rate (*p* = 0.463) and MBL (*p* = 0.393) around implants with internal or external connections.

### Grafting protocol

Out of the 20 included investigations for this analysis, 16 studies used grafting materials [14, 16–20, 23, 24, 26–32] and 4 did not perform any grafting [13–22]. Implant survival was 92.2% with 95% CI (85.1-99.2) for studies that did not graft the gap, while studies that grafted the gap presented with 97.7% with 95% CI (94.3–100). There were no significant differences in survival according to presence or absence of grafting material (*p* = 0.168). Implant success was reported in 6 studies, reporting 83.8% with 95% CI (68.0–99.6) in the graftless group and 97.9% with 95% CI (87.0–100) for the grafted group. There were no statistically significant differences in success according to presence or absence of graft (*p* = 0.150) [13, 16, 20, 22, 28, 30]. Although statistical significance difference was not reached, results showed that grafting favors survival and success rates. With regards to the effect of grafting on MBL, no significant differences were observed between studies using biomaterials 1.39 ± 0.63 mm with 95% CI (0.87–1.92) versus those that did not perform grafting 0.79 ± 0.55 mm. There were no statistically significant differences in MBL according to presence or absence of graft (*p* = 0.333) [13, 16, 18–21, 24, 25, 27, 29, 32].

### Flap/flapless surgery

Fourteen studies reported a flapless technique [2, 3, 13, 14, 17, 20–22, 24–28, 30, 31] while the remaining 6 investigations reported the use of a full thickness flap for implant placement [15, 16, 18, 19, 29, 32]. No significant differences were observed in implant survival (*p* = 0.742), implant success (*p* = 0.932) and MBL. MBL was 1.41± 0.38mm with 95% CI (0.66 2.15) when a flap was elevated for implant placement and 1.19 ±0.34 mm with 95% CI (0.53 1.85) with a flapless approach. Flap elevation also showed no effect on survival and success rates. (*p* = 0.667)

### Implant diameter

Analyzed implants were divided into two groups: < 5 mm [15, 19, 22, 26, 27, 29] and > 5 mm [13, 14, 17, 21, 23, 24, 28, 30] diameter. Implant survival rate for < 5 mm group was 96.1% with 95% CI (88.9–100), and 94.5% with 95% CI (88.2–100) for > 5 mm. MBL was assessed in 6 studies reporting a mean bone loss of 0.74 ± 0.32 mm with 95% CI (0.13–1.35) [19, 27, 29] for > 5 mm group, and 1.41 ± 0.38 mm with 95% CI (0.66–2.15) for < 5 mm group [13, 21, 23], without statistical significant difference (*p* = 0.205).

### Surgical protocol

The influence of surgical protocol on implant survival was assessed in 19 studies showing 97.1% with 95% CI (92.9–100) for implant placed in one surgical stage, in which a healing abutment or immediate restoration has been placed on the day of surgery. For implants placed in 2 surgical phases was 95.3% with 95% CI (89.8–100) [13, 14, 16, 18, 20–22, 25, 28–30, 32]; without statistical significant difference (*p* = 0.616) [15, 19, 23, 24, 26, 27, 31]

MBL was assessed in 11 studies, reporting 1.43 ± 0.92 mm with 95% CI (0.87–1.99) mean MBL for implants placed in one surgical stage [13, 16, 18, 20, 21, 25, 29, 32] and 0.91 ± 0.46 mm with 95% CI (0.01–1.81) mean MBL for implants placed in two stages [19, 24, 27].

### Presence of buccal bone wall

**Fig. 5:**
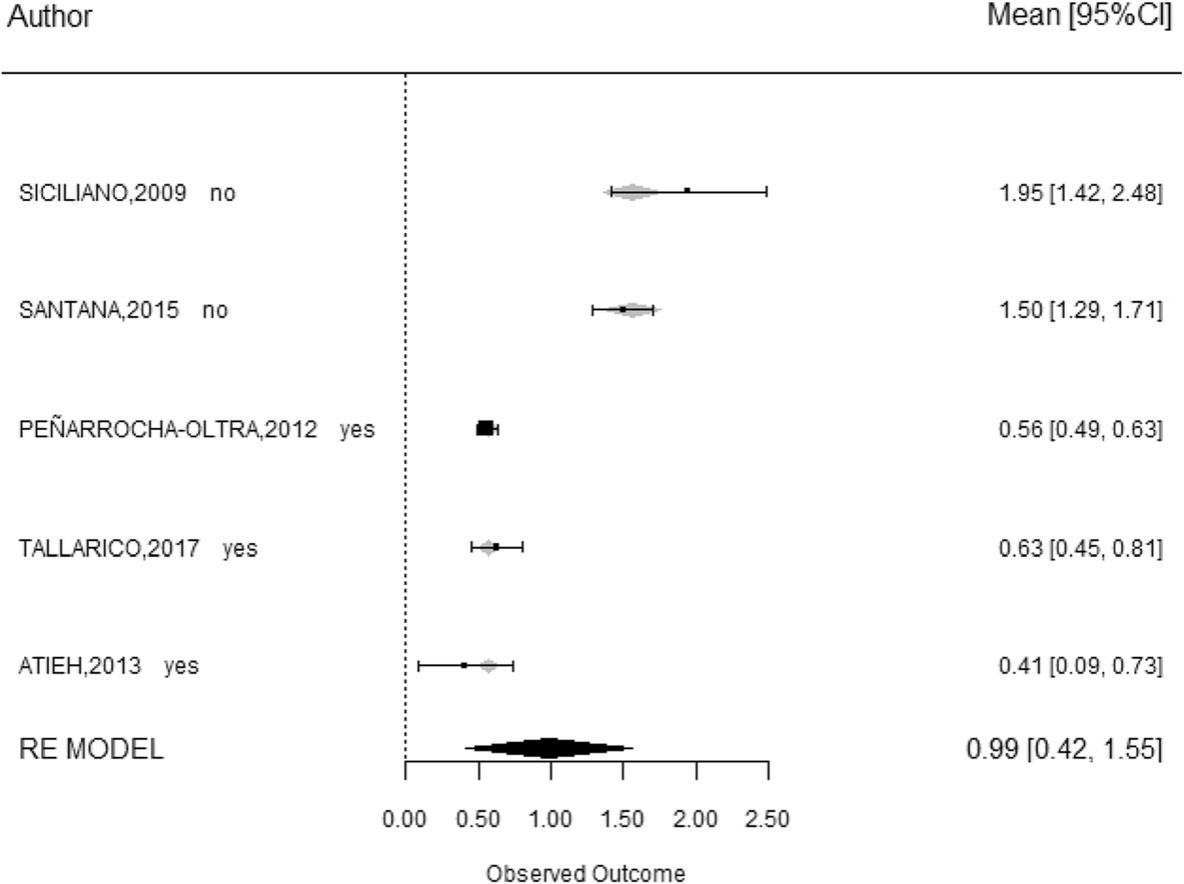
Influence of buccal bone presence on marginal bone loss

Nine studies were included in this analysis, with three reporting the absence of the buccal bone (assessed clinically using a periodontal probe) after tooth extraction [27, 29, 31] and six installing implants only when the buccal bone wall was present [13, 14, 16, 23, 24, 28]. Survival rate was 100% with 95% CI (89.1–100) for studies without buccal bone and 92.1% with 95% CI (84.3–99.9) for studies with buccal bone. There were no significant differences in success according to presence or absence of buccal plate (*p* = 0.247). Five studies analyzed MBL and showed 1.56 ± 0.10 mm with 95% CI (1.37–1.76) when no buccal bone was present [27, 29] and 0.56 ± 0.11 mm with 95% CI (0.79–1.21) MBL when buccal plate was present [13, 16, 24]. There were statistical significant differences in MBL according to the presence or absence of the buccal bone (*p* < 0.001) (Fig. 5).

### Implant loading protocol

Eighteen studies [15–28, 30, 31] reported loading at least 3 months after implant placement, and two studies reported immediate aesthetic restoration placement [13, 14]. The loading protocol significantly influenced survival rates (*p* = 0.007) with immediate loaded implants showing 84.1% with 95% CI (74.6–98.6) survival rate, and studies that used delayed loading protocols showed 97.7% with 95% CI (94.3–100). Implant success and MBL could not be analyzed due to small sample size.

### Meta-analysis

**Fig. 6:**
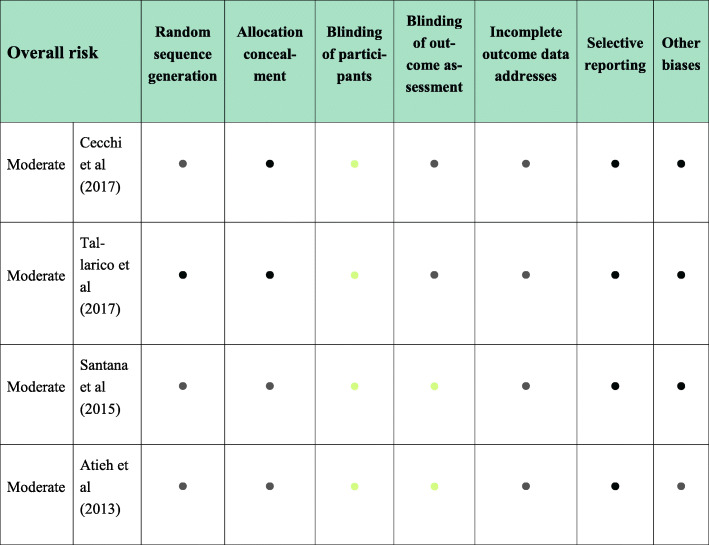
Risk of bias analysis

Four RCTs compared immediate implants versus implants installed in healed molar sites after 6 months from tooth extraction in terms of survival rates and MBL. The risk of bias has been found to be moderate among these investigations (Fig. 6) [13, 23, 24, 27].

### Implant survival rate

**Fig. 7:**
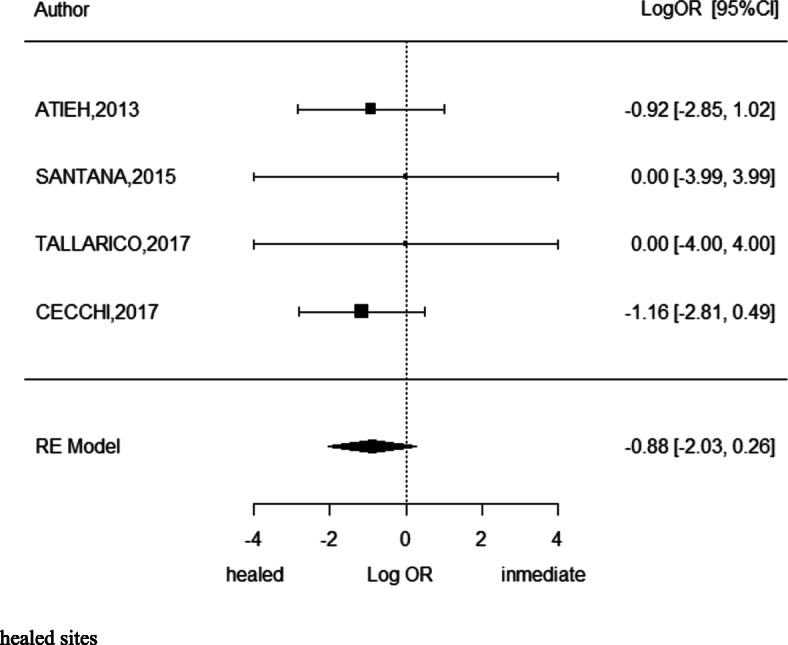
Meta-analysis of implant survival rate between immediate and conventional implants

The overall effect measure of the meta-analysis was OR = 0.41 (95% CI 0.13–1.30) *I*^2^ = 0%; without statistical significant difference (*p* = 0.131). This indicated that the probability of survival with immediate implants was reduced by 59% compared to implants in healed sites (Fig. 7).

### Marginal bone loss

**Fig. 8:**
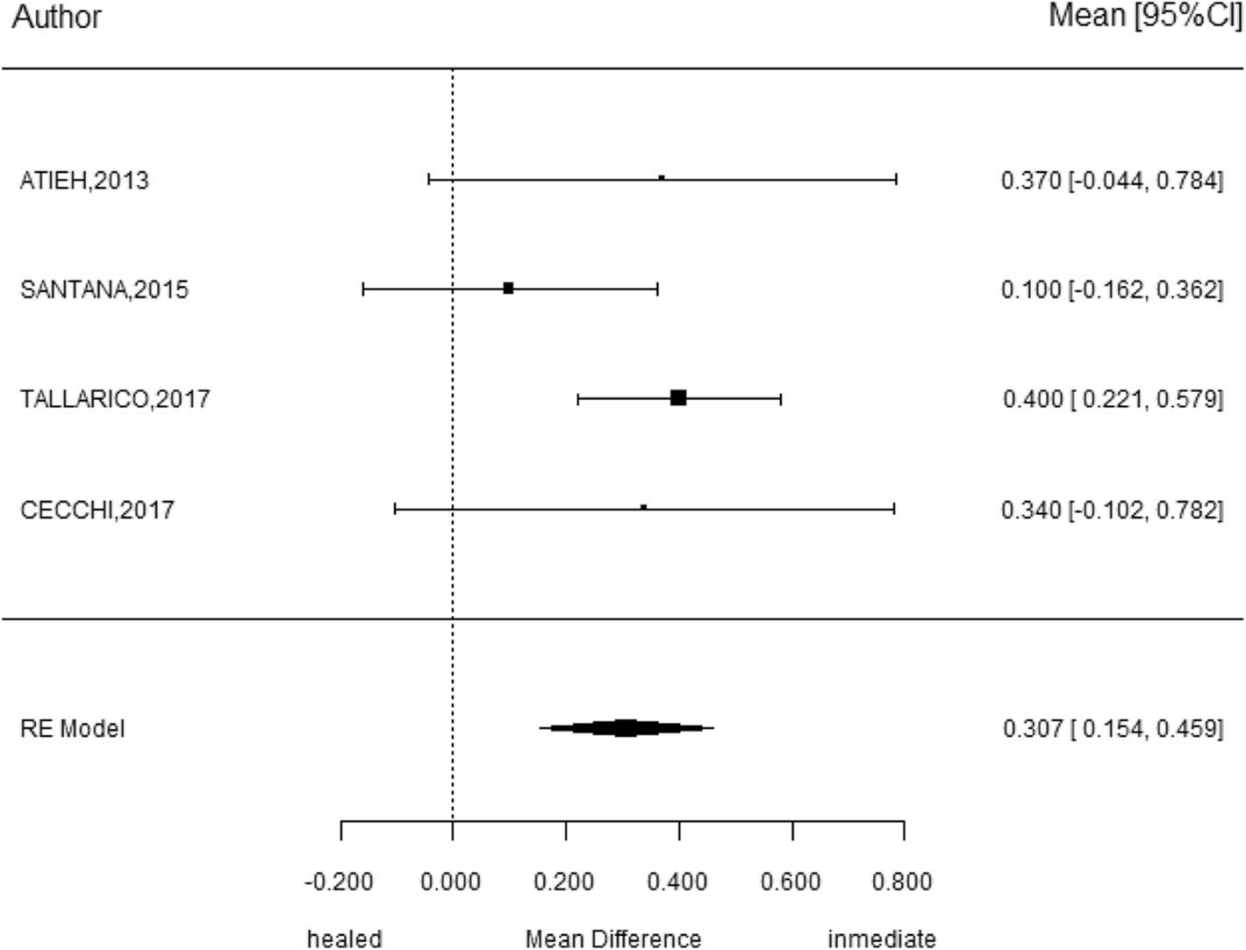
Meta-analysis of marginal bone loss between immediate and conventional implants

Four investigations showed a weighted mean difference of 0.31 ± 0.8 mm with 95% CI (0.15–0.46) finding statistically significant more marginal bone loss at immediate implant placement versus implants in healed sites. (*p* < 0.001) *I*^2^ = 15.2% (Fig. 8).

## Discussion

The purpose of this investigation was to systematically analyze the available literature reporting on the survival and success rates as well as the MBL of implants placed immediately in molar areas. Although a systematic review was previously performed on this topic years ago, implant therapy has rapidly evolved and new evidence has emerged. Hence, this study aimed at updating the previously mentioned review given that multiple studies have been performed on this topic since 2010 [6].

Previous investigations revealed that timing of implant placement plays a critical role in treatment outcomes and that other factors can also strongly influence therapeutic results, including but not limited to implant location, implant connection, smoking, as well as implant-socket gap grafting and flap elevation. These variables potentially influencing treatment outcomes are also analyzed in this review.

This study revealed that implants installed in fresh extraction sockets on molar sites yielded survival rates of 96.6% over a minimum 1 year follow up period. These results are in concordance to those achieved by Lang et al. where immediate implants placed in extraction sockets of anterior teeth yielded a 98.4% survival rate after a 2-year follow up [4]. A successful treatment should be considered when the implant is free of technical and/or biological complications and, in addition, aesthetic outcomes are satisfactory. In this systematic review the average success rate was 93.3%. Eleven studies rendered information on MBL showing an average of 1.29 ± 0.24 mm; these results are comparable with those of a prospective clinical study performed on 30 patients which received immediate implants in the anterior zone, where the mean peri-implant bone loss seen after 3 years of follow-up was 1.00 mm [33]. However, the meta-analysis showed a weighted mean difference of 0.31 ± 0.8 mm; finding statistical significant more marginal bone loss at implants installed immediately (*p* < 0.001). The reported bone loss is probably influenced by a variety of different factors including the resorption process occurring after tooth extraction. This resorption can reach 1.53 mm during the first 12 months [34]. In order to minimize the volumetric changes after tooth extraction, a flapless implant placement is recommended; however, no significant differences were seen in MBL when comparing flap and flapless approaches. These results also coincide with the data reported in a meta-analysis performed by Chcranovic et al. who reported no statistically significant differences in implant failure or MBL around implants placed after flap elevation versus flapless implants placement [35]. Aspects favoring the use of a flapless technique could include minimization of postoperative peri-implant tissue loss, decreased operative time, more rapid post-surgical healing, fewer postoperative complications, and increased patient comfort. Nevertheless, flap elevation can allow the clinician to better visualize the area in which the implant should be installed as well as better access to proper regenerative procedures.

The effect of grafting protocol on MBL also showed no significant differences between studies using biomaterials for the gap, versus those with a graftless approach. In addition, although statistically significance difference was not reached, it appeared that grafting the gap reported higher survival and success rates. A recent study conducted by Tarnow et al. also suggested that grafting the gap at immediate implant sites combined with a contoured healing abutment or a provisional restoration resulted in reduction of ridge contour change. Therefore, it is recommended to graft the gap and use contoured healing abutment or provisional restorations at the time of immediate implant placement [36].

In the present study, the loading protocol also significantly influenced implant survival rates (*p* = 0.007). Immediately loaded implants showed 84.1% survival rate, while the delayed loading protocol showed 97.7%. Conversely, Benic et al. in a recent systematic review concluded that immediately and conventionally loaded single-implant crowns are equally successful regarding implant survival and MBL [37]. Also, Meloni et al. analyzed immediate non-occlusal versus delayed loading of mandibular first molars during a 5-year follow up, finding 100% implant survival rate for both groups; and a mean marginal bone loss of 0.62 ± 0.45 mm in the immediate loading group and 0.69 ± 0.33 mm for the delayed group [38].

Limitations of the present systematic review include the analysis of small sample sizes, heterogeneities in the included investigations, and low number of randomized controlled clinical trial comparing implants installed in healed ridges vs implant immediately placed after extraction. An analysis evaluating the influence of implant position (i.e., mandible vs maxilla) would be interesting in order to elucidate the influence of different anatomical aspects such as the maxillary sinus and/or bone quality, on the survival and success rates of immediate implants. In addition, more RCTs are needed comparing immediate implants versus early implant placement and implants installed in healed ridges. In addition, future investigations should focus on the long-term results of this treatment protocol.

## Conclusions

In selected scenarios, immediate implant in molar extraction sockets might be considered a predictable technique, as demonstrated by a high implant survival and success rates, with minimal MBL. The ideal treatment protocol consists on a flapless approach, a one-stage implant placement, without performing immediate loading, grafting the gap and the use of implants with < 5 mm diameter. More studies are needed focused in the role of implant surfaces, biomaterials in the gap, and the anatomical characteristics of the recipient sites.

## Data Availability

Not applicable

## Abbreviations

IIP: Immediate implant placement
IR: Immediate restorations
MBL: Marginal bone loss
RCTs: Randomized controlled trials
NOS: Newcastle-Ottawa Scale
IC: Confidence interval

## References

[1] Adell R, Lekholm U, Rockler B, Brånemark PI (1981). A 15-year study of osseointegrated implants in the treatment of the edentulous jaw. Int J Oral Surg.

[2] Hämmerle CH, Chen ST, Wilson TG (2004). Consensus statements and recommended clinical procedures regarding the placement of implants in extraction sockets. Int J Oral Maxillofac Implants.

[3] Schulte W, Heimke G (1976). The Tübinger immediate implant. Quintessenz..

[4] Lang NP, Pun L, Lau KY, Li KY, Wong MC (2012). A systematic review on survival and success rates of implants placed immediately into fresh extraction sockets after at least 1 year. Clin Oral Implants Res.

[5] Sanz M, Cecchinato D, Ferrus J, Salvi GE, Ramseier C, Lang NP (2014). Implants placed in fresh extraction sockets in the maxilla: clinical and radiographic outcomes from a 3-year follow-up examination. Clin Oral Implants Res.

[6] Atieh MA, Payne AG, Duncan WJ, de Silva RK (2010). Cullinan MP immediate placement or immediate restoration/loading of single implants for molar tooth replacement: a systematic review and meta-analysis. Int J Oral Maxillofac Implants.

[7] Fugazzotto PA (2008). Implant placement at the time of maxillary molar extraction: treatment protocols and report of results. J Periodontol.

[8] Fugazzotto PA (2008). Implant placement at the time of mandibular molar extraction: description of technique and preliminary results of 341 cases. J Periodontol.

[9] Bianchi AE, Sanfilippo F (2004). Single-tooth replacement by immediate implant and connective tissue graft: a 1-9-year clinical evaluation. Clin Oral Implants Res.

[10] Fugazzotto PA (2005). Treatment options following single-rooted tooth removal: a literature review and proposed hierarchy of treatment selection. J Periodontol.

[11] Fugazzotto PA (2006). Implant placement at the time of maxillary molar extraction: technique and report of preliminary results of 83 sites. J Periodontol.

[12] Fugazzotto PA (2002). Implant placement in maxillary first premolar fresh extraction sockets: description of technique and report of preliminary results. J Periodontol.

[13] Atieh MA, Alsabeeha NH, Duncan WJ, de Silva RK, Cullinan MP, Schwass D, Payne AG (2013). Immediate single implant restorations in mandibular molar extraction sockets: a controlled clinical trial. Clin Oral Implants Res.

[14] Prosper L, Crespi R, Valenti E, Capparé P, Gherlone E (2010). Five-year follow-up of wide-diameter implants placed in fresh molar extraction sockets in the mandible: immediate versus delayed loading. Int J Oral Maxillofac Implants.

[15] Urban T, Kostopoulos L, Wenzel A (2012). Immediate implant placement in molar regions: a 12-month prospective, randomized follow-up study. Clin Oral Implants Res.

[16] Peñarrocha-Oltra D, Demarchi CL, Maestre-Ferrín L, Peñarrocha-Diago M, Peñarrocha-Diago M (2012). Comparison of immediate and delayed implants in the maxillary molar region: a retrospective study of 123implants. Int J Oral Maxillofac Implants.

[17] Peñarrocha-Diago M, Carrillo-Garcîa C, Boronat-Lopez A, García-Mira B (2008). Comparative study of wide-diameter implants placed after dental extraction and implantspositioned in mature bone for molar replacement. Int J Oral Maxillofac Implants.

[18] Cafiero C, Annibali S, Gherlone E, Grassi FR, Gualini F, Magliano A, Salvi GE, ITI study group Italia (2008). Immediate transmucosal implant placement in molar extraction sites: a 12-month prospective multicenter cohort study. Clin Oral Implants Res.

[19] Hamouda NI, Mourad SI, El-Kenawy MH, Maria OM (2015). Immediate implant placement into fresh extraction socket in the mandibular molar sites: a preliminary study of a modified insertion technique. Clin Implant Dent Relat Res.

[20] Hayacibara RM, Gonçalves CS, Garcez-Filho J, Magro-Filho O, Esper H, Hayacibara MF (2013). The success rate of immediate implant placement of mandibular molars: a clinical and radiographic retrospective evaluation between 2 and 8 years. Clin Oral Implants Res.

[21] Hattingh A, Hommez G, De Bruyn H, Huyghe M, Vandeweghe S (2018). A prospective study on ultra-wide diameter dental implants for immediate molar replacement. Clin Implant Dent Relat Res.

[22] Amato F, Polara G (2018). Immediate implant placement in single-tooth molar extraction sockets: a 1- to 6-year retrospective clinical study. Int J Periodontics Restorative Dent.

[23] Checchi V, Felice P, Zucchelli G, Barausse C, Piattelli M, Pistilli R (2017). Wide diameter immediate post-extractive implants vs delayed placement of normal-diameter implants in preserved sockets in the molar region: 1-year post-loading outcome of a randomised controlled trial. Eur J Oral Implantol.

[24] Tallarico M, Xhanari E, Pisano M, Gatti F, Meloni SM (2017). Molar replacement with 7 mm-wide diameter implants: to place the implant immediately or to wait 4 months after socket preservation? 1 year after loading results from a randomised controlled trial. Eur J Oral Implantol.

[25] Chen Y, Yuan S, Zhou N, Man Y (2017). Transcrestal sinus floor augmentation with immediate implant placement applied in three types of fresh extraction sockets: a clinical prospective study with 1-year follow-up. Clin Implant Dent Relat Res.

[26] Wychowanski P, Wolinski J, Kacprzak M, Tomkiewicz W, Bartlomiej I, Szubinska-Lelonkiewicz D (2017). Immediate palatal molar implants: a simple, safe, minimally invasive technique. Int J Periodontics Restorative Dent.

[27] Santana RB, Santana CM, Dibart S (2015). Platelet-derived growth factor-mediated guided bone regeneration in immediate implant placement in molar sites with buccal bone defects. Int J Periodontics Restorative Dent.

[28] Vandeweghe S, Hattingh A, Wennerberg A, Bruyn HD (2011). Surgical protocol and short-term clinical outcome of immediate placement in molar extraction sockets using a wide body implant. J Oral Maxillofac Res.

[29] Siciliano VI, Salvi GE, Matarasso S, Cafiero C, Blasi A, Lang NP (2009). Soft tissues healing at immediate transmucosal implants placed into molar extraction sites with buccal self-contained dehiscences. A 12-month controlled clinical trial. Clin Oral Implants Res.

[30] Jiansheng H, Dongying X, Xianfeng W, Baoyi X, Qiong L, Jincai Z (2012). Clinical evaluation of short and wide-diameter implants immediately placed into extraction sockets of posterior areas: a 2-year retrospective study. J Oral Implantol.

[31] Hu C, Gong T, Lin W, Yuan Q, Man Y (2017). Immediate implant placement into posterior sockets with or without buccal bone dehiscence defects: a retrospective cohort study. J Dent.

[32] Cafiero C, Marenzi G, Blasi A, Siciliano VI, Nicolò M, Sammartino G (2013). Soft and hard tissues healing at immediate transmucosal implants placed into molar extraction sites with collagen membrane uncovered: a 12-month prospective study. Implant Dent.

[33] Cosyn J, Eghbali A, De Bruyn H, Collys K, Cleymaet R, De Rouck T (2011). Immediate single-tooth implants in the anterior maxilla: 3-year results of a case series on hard and soft tissue response and aesthetics. J Clin Periodontol.

[34] Schropp L, Kostopoulos L, Wenzel A, Isidor F (2005). Clinical and radiographic performance of delayed-immediate single-tooth implant placement associated with peri-implant bone defects. A 2-year prospective, controlled, randomized follow-up report. J Clin Periodontol.

[35] Chrcanovic BR, Albrektsson T, Wennerberg A (2014). Flapless versus conventional flapped dental implant surgery: a meta-analysis. PLoS One.

[36] Tarnow DP, Chu SJ, Salama MA, Stappert CF, Salama H, Garber DA (2014). Flapless postextraction socket implant placement in the esthetic zone: part 1. The effect of bone grafting and/or provisional restoration on facial-palatal ridge dimensional change-a retrospective cohort study. Int J Periodontics Restorative Dent.

[37] Benic GI, Mir-Mari J, Hämmerle CH (2014). Loading protocols for single-implant crowns: a systematic review and meta-analysis. Int J Oral Maxillofac Implants.

[38] Meloni SM, Baldoni E, Duvina M, Pisano M, De Riu G, Tallarico M (2018). Immediate non-occlusal versus delayed loading of mandibular first molars. Five-year results from a randomised controlled trial. Eur J Oral Implantol.

